# Adenovirus-mediated downregulation of the ubiquitin ligase RNF8 sensitizes bladder cancer to radiotherapy

**DOI:** 10.18632/oncotarget.6909

**Published:** 2016-01-13

**Authors:** Mei-Jun Zhao, Yan-Feng Song, Hai-Tao Niu, Ying-Xia Tian, Xu-Guang Yang, Kun Xie, Yu-Hong Jing, De-Gui Wang

**Affiliations:** ^1^ Department of Anatomy and Histology, School of Basic Medical Sciences, Lanzhou University, Lanzhou, China; ^2^ Department of Internal Medicine, Gansu Provincial Academic Institute for Medical Research, Lanzhou, China; ^3^ Department of Urology, Affiliated Hospital of Qingdao University, Qingdao, China

**Keywords:** bladder cancer, adenovirus, RNF8, radiotherapy, gene therapy

## Abstract

The ubiquitin ligase RNF8 promotes the DNA damage response (DDR). We observed that the expression of RNF8 was increased in bladder cancer cells and that this change in RNF8 expression could be reversed by adenovirus-mediated shRNA treatment. Moreover, we found that RNF8 knockdown sensitized bladder cancer cells to radiotherapy, as demonstrated by reduced cell survival. Additionally, the absence of RNF8 induced a high rate of apoptosis and impaired double-strand break repair signaling after radiotherapy. Furthermore, experiments on nude mice showed that combining shRNF8 treatment with radiotherapy suppressed implanted bladder tumor growth and enhanced apoptotic cell death *in vivo*. Altogether, our results indicated that RNF8 might be a novel target for bladder cancer treatment.

## INTRODUCTION

Bladder cancer is the second most frequent cause of mortality among cancers of the genitourinary system and is the sixth most common malignancy in men worldwide [1]. Some frequently used therapeutic strategies, such as radical cystectomy (RC), may be associated with significant morbidity and may affect patients’ comfort and quality of life [2-4], and the 5-year recurrence-free survival rate associated with bladder cancer treatments remains poor [5-8]. Other conventional bladder-sparing treatments, such as transurethral resection, radiotherapy and chemotherapy, also provide limited effects [9-14]. Therefore, the dismal clinical context of bladder cancer necessitates the development of new therapeutic strategies.

The clinical application of adenovirus-mediated gene therapy displays the potential to substantially improve the therapeutic outcomes of patients, along with the possibility to preserve the bladder [15-19]. Recombinant adenovirus is an efficient gene delivery system in which the virus replicates episomally without host genome insertion [20]. In this study, we employed a special replication-deficient adenovirus type 5 (Ad5), which can minimize the danger of host genome instability and tumorigenesis [21]. Moreover, Ad5 can transfer genes into both dividing and non-dividing cells. As a result, this virus is suitable for malignancies with a low mitotic index [21].

We observed that RNF8 was highly expressed in bladder cancer cells. Thus, we hypothesized that RNF8 might be a potential target for bladder cancer treatment. Cells derived from RNF8−/− mice exhibit higher radiosensitivity than wild-type mice [22]. Correspondingly, high expression of RNF8 might reduce the efficacy of radiotherapy for bladder cancer. RNF8 activates a cascade of protein modifications and localization alterations upon double-strand break (DSB) induction [23-30], which can be generated by ionizing radiation (IR) [31-33], to repair damaged DNA. The DNA damage response (DDR) must be silenced in the setting of cancer treatment because DNA damage repair defects may cause hypersensitivity to genotoxic agents [34].

Here, we explored the specific effects of RNF8 knockdown via adenovirally delivered shRNA on the response to radiotherapy. Although current anti-cancer virotherapy has clinical limitations, as systemically administered adenoviruses can elicit a host immune response [21], the bladder is an anatomically accessible organ, rendering it an ideal site for direct intravesicular administration of therapeutic agents [35]. Approximately 80% of bladder cancer patients have non-muscle-invasive bladder cancer (NMIBC) or carcinoma in situ (CIS) at the time of initial presentation [36], so bladder irrigation may be clinically effective for adenoviral vector delivery. Furthermore, for the remaining 20% of newly diagnosed patients with muscle-invasive bladder cancer (MIBC), adenovirus could be injected into bladder tumors extending into the muscular layer under direct visualization using a cystoscope.

## RESULTS

### RNF8 is highly expressed and participates in the DNA damage response in bladder cancer cells

**Figure 1:**
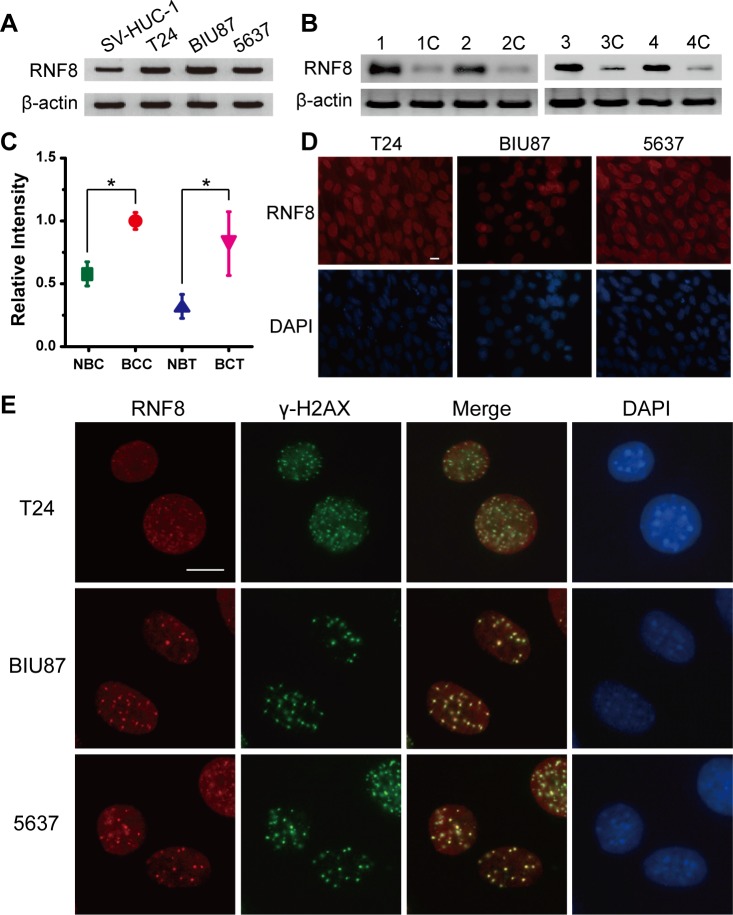
RNF8 is highly expressed in bladder cancer cells and accumulates at DSB sites **A.** Western blotting analysis of T24, BIU87, 5637 and SV-HUC-1 cell lines for RNF8. **B.** Western blotting analysis of bladder cancer patient carcinoma and normal control bladder tumor-adjacent tissues for RNF8. The numbers 1 to 4 and 1C to 4C represent the four randomly selected bladder cancer samples and their controls, respectively. **C.** Quantitative analysis of the Western blotting bands corresponding to RNF8. NBC: normal bladder cells; BCC: bladder cancer cells; NBT: normal bladder tissues; BCT: bladder cancer tissues. * indicates statistical significance compared to the control (*P* < 0.05). **D.** Immunofluorescence staining for RNF8 in the T24, BIU87, and 5637 cell lines. Cells were stained using an anti-RNF8 antibody and counterstained with DAPI. Scale bar, 5 μm. **E.** After irradiation (2 Gy), the cells were fixed, stained using anti-RNF8 and anti-γ-H2AX antibodies and counterstained with DAPI. Scale bar, 5 μm.

To investigate whether RNF8 is spontaneously expressed in bladder cancer cells, we first analyzed three urothelial carcinoma cell lines, T24, BIU87 and 5637, as approximately 90% of malignant bladder tumors are urothelial cell carcinomas [36]. We cultured and harvested cells in the logarithmic phrase of growth for Western blotting analysis. The results showed that the expression of RNF8 in the three bladder cancer cell lines was upregulated compared to the control urothelial cell line SV-HUC-1 (Figure 1A). RNF8 expression was also higher in tissue specimens from bladder cancer patients who underwent radical cystectomy than in normal control bladder cancer-adjacent tissues (Figure 1B). Next, quantitative analysis of the intensity of the Western blotting bands was performed. The average relative intensity of RNF8 was 1.75-fold higher in the T24, BIU87 and 5637 cells than in the SV-HUC-1 cells and was 2.56-fold higher in the bladder cancer tissues than in the tumor-adjacent control tissues (Figure 1C).

Next, we used an immunofluorescence staining assay to detect endogenous RNF8 expression in bladder cancer cells. We observed that RNF8 was diffusely and highly expressed in the cell nucleus (Figure 1D). Then, we evaluated the presence of ionizing radiation-induced foci (IRIF) of RNF8 and γ-H2AX, a marker of DSBs [34], in response to DNA damage after exposure to 2 Gy of X-ray irradiation. The results showed that RNF8 clearly formed IRIF that colocalized with γ-H2AX IRIF (Figure 1E); this finding suggested that RNF8 participated in DNA damage repair after radiotherapy.

### RNF8 knockdown sensitizes bladder cancer cells to radiotherapy

**Figure 2:**
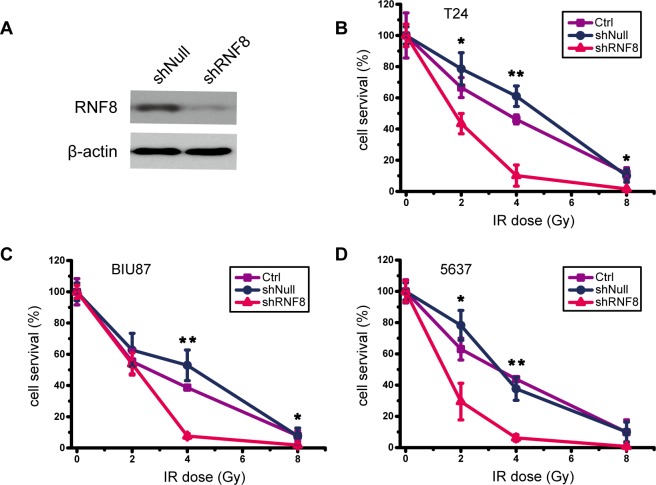
Knockdown of RNF8 impairs the radioresistance of bladder cancer cells **A.** Western blotting for RNF8 expression after shRNF8 transfection. shNull, transfected with empty vector; shRNF8, transfected with the shRNF8-harboring vector. **B.**-**D.** Colony formation assay of cells after exposure to different doses of IR. T24, BIU87 and 5637 cells transfected with the empty adenoviral vector or the shRNF8-harboring adenoviral vector were irradiated at the indicated doses. The numbers of colonies formed in each plate were normalized to the number of colonies in the empty vector-infected, untreated cells, and all experiments were performed in triplicate. Error bars, SD. * indicates statistical significance compared to the control (*P < 0.05, **P < 0.01).

The enhancement of the DDR can promote cancer cell survival and growth by sustaining genomic integrity; thus, DDR activity likely confers therapeutic resistance. Conversely, loss-of-function alterations of the DDR may confer therapeutic sensitization [37, 38]. Because RNF8 was highly expressed in bladder cancer cells and participated in the DDR after radiotherapy (Figure 1), to determine the extent of the contribution of RNF8 to radioresistance, we knocked down RNF8 expression using adenovirus-mediated shRNA in bladder cancer cell lines and then performed a colony formation assay. The efficient downregulation of RNF8 was confirmed by Western blotting (Figure 2A). The data from the colony formation assay showed that treatment with several doses of IR (2, 4 and 8 Gy) did not appear to exert differential effects between untreated and shNull-treated control cells at any IR dose; in contrast, adeno-shRNF8-treated cells displayed a decreased survival rate compared to their controls (Figure 2B, 2C, 2D). Altogether, these results suggested that the downregulation of RNF8 sensitized bladder cancer cells to radiotherapy.

### RNF8 knockdown increases IR-induced apoptosis in bladder cancer cells

**Figure 3:**
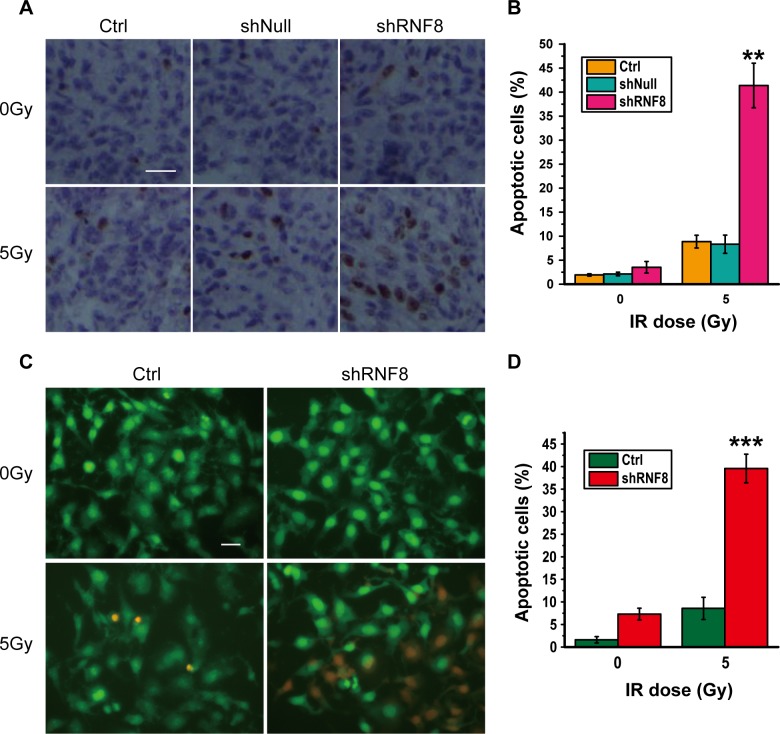
Knockdown of RNF8 leads to increased IR-induced apoptosis in bladder cancer cells **A.** TUNEL assay of T24 cells. The cells were transfected with empty vector or the shRNF8-harboring vector and were exposed to the indicated doses of IR. Scale bar, 50 μm. **B.** Quantification of TUNEL-positive cells among the T24 cells. An average of 20 fields were counted at 60&times; magnification. The data are representative of 3 different experiments. * indicates statistical significance compared to the control (**P < 0.01). **C.** AO/PI double staining of T24 cells. The cells were transfected with the shRNF8-harboring vector and exposed to the indicated doses of IR. Scale bar, 10 μm. **D.** Quantification of PI-positive cells among the T24 cells. The data are representative of 3 different experiments. * indicates statistical significance compared to the control (***P < 0.001).

To determine the underlying mechanisms by which RNF8 knockdown conferred radiation sensitivity, immunocytochemistry was performed on the T24 bladder cancer cell line for a TUNEL assay. shRNF8-treated cells and their controls were either untreated or irradiated (5 Gy). The non-irradiated bladder cancer cells displayed low levels of apoptosis. However, after exposure to 5Gy IR, T24 cells treated with shRNF8 showed a significant increase in the proportion of TUNEL-positive cells compared to the control cells (Figure 3A, 3B). The data from 5637 and BIU87 cells were similar to those from T24 cells (data not shown). Additionally, Acridine orange (AO)/propidium iodide (PI) double staining was performed to further investigate the apoptosis of T24 cells upon treatment with shRNF8 and IR, and the results were consistent with those of the TUNEL assay (Figure 3C, 3D). Therefore, downregulation of RNF8 resulted in enhanced IR-induced apoptotic cell death, and this finding suggested that combination treatment of bladder cancer cells with shRNF8 and IR can substantially improve the efficacy of radiotherapy by inducing apoptosis.

### RNF8 knockdown disrupts the DNA damage repair pathway in bladder cancer cells

**Figure 4:**
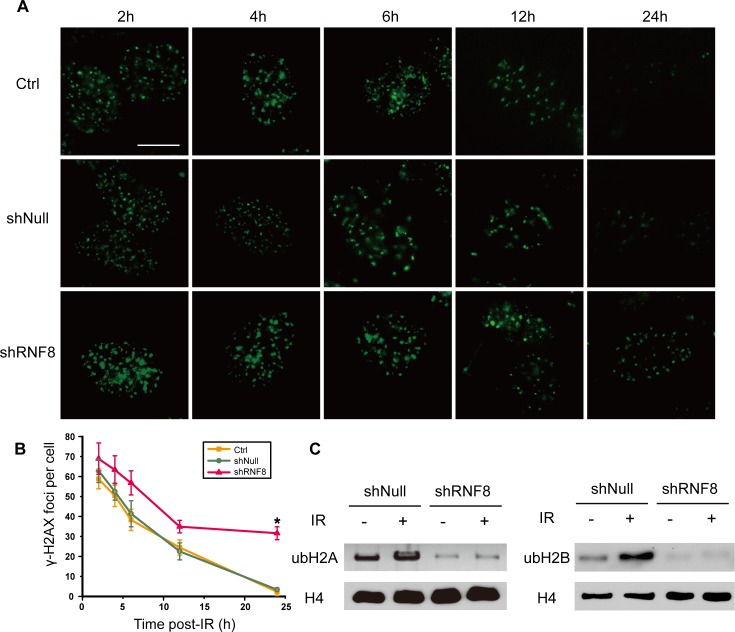
shRNF8 disrupts the DDR post-irradiation in bladder cancer cells **A.** The formation of γ-H2AX foci was observed by fluorescence microscopy. T24 cells transfected with empty vector or the shRNF8-harboring vector were irradiated with 5 Gy X-ray irradiation and then allowed to recover for the indicated number of hours. Afterwards, the cells were fixed and stained using an anti-γ-H2AX antibody. Scale bar, 5 μm. **B.** The numbers of γ-H2AX foci at the indicated time points post-irradiation. At least 100 cells were quantified for each experiment. The data are presented as the means&plusmn;SD of at least 3 independent experiments. * indicates statistical significance (*P < 0.05; Student's *t*-test). **C.** T24 cells with or without RNF8 knockdown were treated with IR (5Gy) or were untreated. Then, the cells were analyzed by Western blotting for Ub-H2A and Ub-H2B expression. H4 was used as a loading control.

To explore whether RNF8 participated in the DDR in bladder cancer cells, we utilized T24 cells to examine the process of post-irradiation DNA damage repair in the presence or absence of RNF8 knockdown by detecting γ-H2AX foci at different time points. Cells infected with adenovirus-mediated shRNF8 or shNull vectors and control cells were treated with 5 Gy IR. No difference in the number of γ-H2AX IRIF was observed between the three groups at early time points (2 and 4 hours) post-IR. In contrast, at 24 hours post-IR, when most DSBs had been repaired in untreated and shNull-treated control cells, the number of γ-H2AX IRIF in shRNF8 cells was significantly greater than that in control cells (Figure 4A, 4B). Thus, we demonstrated that RNF8 was crucial for IR-induced DNA damage repair in bladder cancer cells.

To investigate the impact of RNF8 on H2A and H2B ubiquitination in bladder cancer cells, T24 cells transfected with shRNF8 or shNull were exposed to 5 Gy IR. Then, we prepared cell lysates for Western blotting. The levels of both Ub-H2A and Ub-H2B were increased after IR exposure in control cells; however, the Ub-H2A and Ub-H2B levels were decreased in RNF8-silenced cells compared with their mock shNull-treated controls regardless of exposure to IR. The results of this analysis showed that in bladder cancer cells, RNF8 participated in the radiotherapy-induced DNA damage response via the ubiquitination of H2A and H2B.

### RNF8 knockdown impairs the recruitment of 53BP1, BRCA1 and RAP80 to DNA damage sites

**Figure 5:**
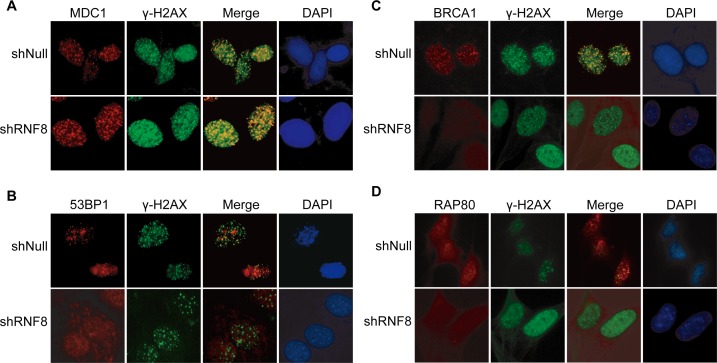
RNF8-related factors in the DSB repair pathway **A.**-**D.** T24 cells transfected with shRNF8 or shNull were mock-treated (0 Gy) or irradiated (5 Gy). The cells were fixed 1 h post-IR and stained with anti-MDC1 (A), anti-53BP1 (B), anti-BRCA1 (C) or anti-RAP80 (D) antibodies and counterstained with DAPI. All experiments were independently repeated multiple times.

RNF8 controls the cellular responses to genotoxic stress by regulating the interactions, localization, and function of DDR proteins [23]. To further investigate the role of RNF8 in DNA damage repair in bladder cancer cells, we depleted RNF8 using shRNA and examined whether the formation of IRIF of various DNA damage signaling/repair proteins was RNF8-dependent. We first used the T24 cell line to examine MDC1 foci, as MDC1 phosphorylation is required for the binding of RNF8 to DSB sites [30]. The results showed no significant differences in MDC1 IRIF between the control cells and the shRNF8-treated cells after IR exposure (5 Gy), as both groups clearly displayed MDC1 IRIF at DNA damage sites (Figure 5A).

Next, we evaluated 53BP1 IRIF formation in the absence of RNF8 in bladder cancer cells. We observed that 53BP1 accumulated at DNA damage sites in control cells but that 53BP1 displayed only small, transient foci at early time points (0.5 hour) that were not retained at DNA damage sites in shRNF8 cells; the 53BP1 foci were diffusely distributed in the nuclei but did not clearly form IRIF (Figure 5B).

In addition to 53BP1, BRCA1 plays a critical role in homologous recombination (HR)-mediated DNA repair, and its binding protein, RAP80, which contain an ubiquitin-interacting motif (UIM), is required for the optimal accumulation of BRCA1 at DNA damage sites in response to IR [39]. Our results showed that downregulation of RNF8 impaired the recruitment of BRCA1 and RAP80 to DNA damage sites in bladder cancer cells (Figure 5C, 5D). Collectively, these results indicated that in RNF8-silenced bladder cancer cells, γ-H2AX and MDC1, which function upstream of RNF8, clearly formed IRIF after radiotherapy. However, the downstream DNA repair proteins 53BP1, BRCA1 and RAP80 displayed abrogated recruitment and retention at DSB sites. These observations suggested that the DSB signaling and repair cascade was defective in the absence of RNF8.

### RNF8 knockdown enhances the outcomes of radiotherapy *in vivo* in malignant bladder tumor models

**Figure 6:**
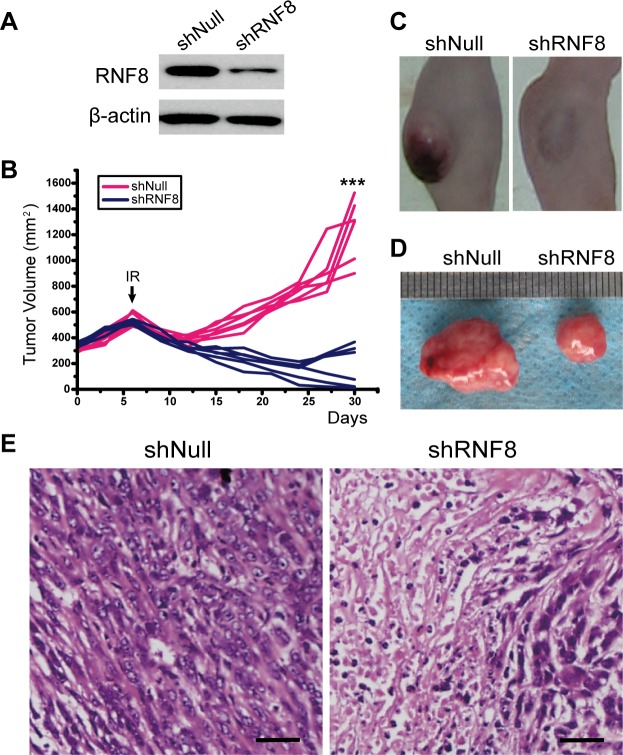
The effect of RNA interference to silence RNF8 on implanted T24 cell-based tumors in nude mice **A.** Western blotting for RNF8 expression after injection of adenovirus *in vivo*. shNull, transfected with empty vector; shRNF8, transfected with the shRNF8-harboring vector. **B.** Growth curve of the subcutaneously implanted tumors in nude mice. The tumor size was compared between each group. A significant decrease in tumor volume in the shRNF8 group was observed due to IR. The arrow indicates the time point of IR treatment. **C.** Post-treatment photographs of nude mice injected with T24 cells followed by shNull- or shRNF8-harboring adenovirus administration combined with radiotherapy. **D.** Photographs of the subcutaneous tumors excised from the nude mice shown in B. The tumor sizes were measured and compared. **E.** H&E staining of the implanted tumors after IR treatment. Tumors from the shRNF8 group showed more necrosis and apoptosis than those from the shNull group. Scale bar, 50 μm.

Because RNF8 knockdown sensitized bladder cancer cells to radiotherapy *in vitro*, we further performed in vivo experiments to explore whether this effect of RNF8 could be observed in animal models. Tumors were established via subcutaneous injection of T24 cells into nude mice. When palpable tumors were established, the mice were randomized into shRNF8- and shNull-treated groups. Adenoviruses were injected into the tumors 3 times, on the 1^st^, 3^rd^ and 5^th^ days after tumor establishment. Western blots were performed to assess RNF8 expression in the tumor samples from the two groups on the 5^th^ day; the results revealed that RNF8 expression was decreased in the shRNF8-treated group compared to the shNull-treated group (Figure 6A). Then, the mice were exposed to IR on the 6^th^ day, and tumor growth was monitored every three days (Figure 6B). In this experiment, tumor growth in the shRNF8-treated group was dramatically decreased compared to the shNull-treated control group, though there was no significant difference in body size or weight between the two groups (Figure 6C, 6D).

Additionally, we expanded these experiments to assess the therapeutic responses of the implanted tumors using histological staining with hematoxylin-eosin (H&E). We observed that the tumor tissue from the shRNF8-treated group showed visible necrosis and apoptosis compared to the shNull-treated control group (Figure 6E). Overall, these results indicated that adenovirus-mediated RNF8 knockdown sensitized bladder cancer cells to radiotherapy *in vivo*.

## DISCUSSION

At present, radiotherapy is an important strategy for cancer treatment, but its results remain far from satisfactory. IR for cancer treatment primarily induces DNA damage to target cells, and this DNA damage is repaired by both HR and nonhomologous end joining (NHEJ) [40]. Correspondingly, the downstream DDR proteins promoted by RNF8 can trigger DSB repair through the HR and NHEJ pathways [24]. Our data showed that RNF8 was highly expressed in bladder cancer cells (Figure 1A, 1B, 1C) and that RNF8 accumulated at DSB sites after radiotherapy (Figure 1D, 1E). As RNF8 performs a pivotal function in the response to DSBs [23], we hypothesized that the upregulation of RNF8 might result in the resistance of bladder cancer to radiotherapy. We can take advantage of the cellular genetic defects associated with the repair mechanisms of DNA lesions induced by genotoxic anti-cancer therapies [34]. Therefore, a potential anti-cancer target might exert its effect by suppressing the functional expression of RNF8. We knocked down RNF8 using an adenovirus-mediated shRNA (Figure 2A) and detected decreased cancer cell survival post-IR following RNF8 knockdown (Figure 2B-2D). Furthermore, our data showed increased apoptosis and an impaired DDR pathway signaling in shRNF8-transfected bladder cancer cells after exposure to 5 Gy IR (Figure 3 and Figure 4A, 4B). Impairment of the histone ubiquitination pathway appears to be the underlying mechanism of radiosensitization. RNF8 ubiquitinates the chromatin components H2A and H2B at the flanking region of DSBs. Once ubiquitinated, Ub-H2A and Ub-H2B facilitate the recruitment of certain downstream DDR factors, such as 53BP1 and BRCA1, to DSB sites [25, 30, 41]. As a result, the ubiquitination of H2A and H2B is important for DSB repair, which may provide radioresistance. We demonstrated that knockdown of RNF8 blocked the histone ubiquitination process (Figure 4C) and blocked ability of a series of downstream DDR factors to clearly form IRIF, which constitute the DNA damage repair cascade (Figure 5). Studies have revealed that lung cancer patients expressing low levels of both BRCA1 and 53BP1 experienced better prognostic outcomes from chemotherapy [42], and our data imply that downstream DDR-related proteins such as 53BP1 and BRCA1 also display potential as a complementary target to treatment with certain genotoxic anti-cancer agents. Altogether, our findings indicated that RNF8 knockdown attenuated radioresistance and enhanced cancer cell death in both *in vitro* bladder cancer cell lines and *in vivo* malignant bladder tumor models established in nude mice (Figure 6). Of note, the results of *in vivo* histological H&E staining in the shRNF8-treated group were consistent with the high apoptosis rate exhibitedby shRNF8-treated bladder cancer cells *in vitro* (Figure 3 and Figure 6E). Our study supports the use of combination therapy for bladder cancer patients based on the specific cellular DDR pathway activated in individual tumors. In addition to its important role in the response to DSBs, RNF8 was also found to elicit telomere protection by ubiquitinating and stabilizing Tpp1 at telomere ends [43]. At the same time, telomerase is activated in the majority of human cancers, and telomerase activation serves to stabilize telomeres and maintain tumor proliferation [44, 45]. Thus, knockdown of RNF8 may suppress bladder cancer cell survival and progression through other supplementary pathways.

There are various advantages to the use of adenovirus-mediated shRNF8 transfection combined with radiotherapy to treat bladder cancer, as this treatment strategy can significantly improve radiosensitivity with bladder preservation. However, there are still some disadvantages to its use. On the one hand, although certain reconstructed adenoviruses specifically targeting bladder cancer have already been invented, the lack of validation of these adenoviruses in the appropriate patient populations and in specific contexts precludes their clinical implementation [16, 46-48]. On the other hand, targeted therapy itself is not suitable in all circumstances, especially when the targeted factor is expressed by and functions in all normal somatic cells. To address these problems, the optimization of combination therapy for bladder cancer, including the invention of bladder-specific vectors and the improvement of bladder irrigation methods for targeted drug delivery, is necessary. Furthermore, because adenovirus can be cleared relatively easily by the immune system, the current technology cannot use an adenovirus-mediated gene delivery system to treat metastatic bladder cancer. In fact, over 70% of patients with NMIBC or CIS experience at least one instance of disease recurrence and progression after successful initial treatment [49, 50], and patients with MIBC generally also experience a poor outcome, as more than 50% of these patients die due to their disease within 5 years despite systemic therapy [51]. As a result, our study aimed to improve the therapeutic efficacy of radiotherapy by disrupting the DDR pathway in tumor cells to ultimately increase the radiosensitivity of bladder cancer. Moreover, radiotherapy itself is a spatially confined therapeutic strategy that provides the possibility of organ preservation.

If RNF8 is also upregulated in other cancer types and if the affected organ is also anatomically accessible, such as the stomach in gastric cancer, therapeutic adenoviral vectors can be perfused or injected under direct visualization using an endoscope. This method could avoid the reduction in the effective drug concentration caused by intravenous administration. Additionally, if other genotoxic anti-cancer agents, such as certain classes of chemotherapeutic agents, eliminate cancer cells via similar molecular mechanisms, knockdown of RNF8 may hypersensitize target cells to the anti-cancer treatment. Future studies will facilitate the development of combination therapies for bladder cancer.

## MATERIALS AND METHODS

### Cell lines and cell culture

The T24, BIU87, and 5637 cell lines were cultured in high-glucose DMEM supplemented with 10% fetal bovine serum and 100 U/ml penicillin/streptomycin (Invitrogen) at 37&deg;C in a humidified 5% CO_2_ chamber.

### Western blotting

Equal amounts of cell lysates were loaded on 10% or 13% polyacrylamide gels and transferred to a PVDF membrane. The detection of proteins was performed using primary antibodies against RNF8 (Abcam, ab105362), β-actin (Abcam, ab129348), Ub-H2A (Merck Millipore, ABE569), Ub-H2B (Merck Millipore, MABE453), and H4 (Abcam, ab51997) and HRP-conjugated anti-rabbit or anti-rat secondary antibodies (Abcam, ab6721, ab6728). Densitometry was performed using Photoshop CC.

### RNF8 depletion via adenovirus-mediated RNA interference

T24, BIU87 and 5637 cells were infected with adenovirus-mediated vectors expressing shRNF8 or shNull; RNF8 knockdown was accomplished using the sequence 5′-ACATGAAGCCGTTATGAAT-3′, and shNull consisted of the empty adenoviral vector (GenePharma). Transfection of the cells with virus was performed according to the manufacturer's instructions.

### Colony formation assay

T24, BIU87, and 5637 cells were transfected with or shRNF8-harboring adenovirus or an empty vector and were incubated for 48 hours. Then, the cells were seeded in 6-well plates at 1,000 cells per dish immediately following irradiation. After culturing for 12 days, the percentage of surviving cells was calculated by comparing the number of colonies formed in the non-irradiated control cultures.

### Immunocytochemical analysis of apoptosis

T24 cells cultured on Snapwell membranes (5 μm) were transfected with shNull or shRNF8 vector and then either untreated or irradiated (5 Gy). After recovering for 24 h, the cells were fixed in Methacarn solution, embedded in paraffin, deparaffinized, rehydrated, and treated with 3% H_2_O_2_ for 1 min. Subsequently, the cells were subjected to TUNEL staining for 2 h, incubated for 1 h with polyclonal HRP-conjugated anti-rabbit IgG (Abcam, ab6721), reacted with DAB, and counterstained with hematoxylin for 2 min. The samples were washed 3 times with phosphate-buffered saline (PBS) containing 0.05% Tween-20 between each step and in distilled water after the final counterstaining step. Images were viewed and acquired using a 60&times; oil objective lens on a Zeiss microscope controlled by AxioVision 4.8 software. All steps were performed at room temperature. At least 100 morphologically intact cells were examined.

### AO/PI double staining for the detection of apoptosis

T24 cells cultured in 6-well plates were transfected with shRNF8 vector or non-transfected and then either untreated or irradiated (5 Gy). After recovering for 24 h, the cells were trypsinized and washed 3 times with PBS. The harvested cells were resuspended in PBS and double stained with 10 &micro;g/ml AO and PI. Images were viewed under a Zeiss fluorescence microscope. At least 100 morphologically intact cells were examined.

### Immunofluorescence analysis of intranuclear focus formation

T24 cells (7&times;10^5^ cells/ml) transfected with shNull or shRNF8 vector were plated on glass coverslips and exposed to X-ray irradiation (5 Gy) or mock-treated (0 Gy) for 1 h. Then, the cells were fixed with 4% paraformaldehyde for 15 min. After blocking with 3% bovine serum albumin/PBS containing Tween-20, the fixed cells were incubated overnight with specific antibodies and washed twice with PBS. Next, the cells were incubated with secondary antibodies for 30 min, washed twice with PBS and counterstained with 1 μg/ml DAPI (Invitrogen). The primary antibodies used were as follows: anti-γ-H2AX (Abcam, ab22551), anti-MDC1 (Abcam, ab11169), anti-53BP1 (Abcam, ab21083), anti-BRCA1 (Abcam, ab16780) and anti-RAP80 (Abcam, ab124763). Images were viewed and acquired using a 60X oil objective lens on a Zeiss fluorescence microscope controlled by AxioVision 4.8 software. All steps were performed at room temperature. At least 100 morphologically intact cells were examined.

### BALB/c nude mice and tumor implantation

Six-week-old athymic BALB/c nude male mice weighing approximately 20-24 g were obtained from the Model Animal Research Center of Nanjing University. Mice were quarantined for a minimum of 5 days in the SPF-Grade Animal House under a 12 h light/dark photoperiod at 24&deg;C. Guidelines concerning animal handling were followed. Tumors were established via subcutaneous (s.c.) injection of T24 cells (1 &times; 10^6^/100 μl). T24 cells were gently resuspended in 100 &micro;l of PBS (pH 7.4; BioSource, Rockville, MD) mixed 1:1 with Matrigel (BD Biosciences, Palo Alto, CA) and injected into the right flank of the mice. Once the tumors reached a size of 100-150 mm^3^, the mice were randomized into control and treatment groups (6 mice per group). Adenoviruses were injected into the tumors 3 times on days 1, 3 and 5 after tumor establishment. Then, the mice were treated with IR, which was delivered using a Pantak Seifert 320 X-ray system at 200 cGy/min (source to skin distance of 50 cm). The mice were lightly sedated with ketamine (0.1 mg/g) and xylazine (0.02 mg/g). Only the tumor, the surrounding skin and subcutaneous tissues were exposed using a specialized lead jig. Tumor volumes, based on caliper measurements, were calculated at intervals of three days according to the formula described by Kim et al. [52].

### H&E staining of tumor tissues

Paraformaldehyde solution-fixed tumor tissues were embedded in paraffin and sliced into 5-μm sections. The sections were stained with H&E. Images were viewed in a 20&times; field using a Nikon microscope and were acquired using Image-Pro Plus version 6.2 software (Media Cybernetics).

## References

[1] Ferlay J, Soerjomataram I, Dikshit R, Eser S, Mathers C, Rebelo M, Parkin DM, Forman D, Bray F (2015). Cancer incidence and mortality worldwide: sources, methods and major patterns in GLOBOCAN 2012. Int J Cancer.

[2] Shabsigh A, Korets R, Vora KC, Brooks CM, Cronin AM, Savage C, Raj G, Bochner BH, Dalbagni G, Herr HW, Donat SM (2009). Defining early morbidity of radical cystectomy for patients with bladder cancer using a standardized reporting methodology. European urology.

[3] Donat SM, Shabsigh A, Savage C, Cronin AM, Bochner BH, Dalbagni G, Herr HW, Milowsky MI (2009). Potential impact of postoperative early complications on the timing of adjuvant chemotherapy in patients undergoing radical cystectomy: a high-volume tertiary cancer center experience. European urology.

[4] Gakis G, Efstathiou J, Lerner SP, Cookson MS, Keegan KA, Guru KA, Shipley WU, Heidenreich A, Schoenberg MP, Sagaloswky AI, Soloway MS, Stenzl A (2013). ICUD-EAU International Consultation on Bladder Cancer 2012: Radical cystectomy and bladder preservation for muscle-invasive urothelial carcinoma of the bladder. European urology.

[5] Stein JP, Lieskovsky G, Cote R, Groshen S, Feng AC, Boyd S, Skinner E, Bochner B, Thangathurai D, Mikhail M, Raghavan D, Skinner DG (2001). Radical cystectomy in the treatment of invasive bladder cancer: long-term results in 1,054 patients. J Clin Oncol.

[6] Madersbacher S, Hochreiter W, Burkhard F, Thalmann GN, Danuser H, Markwalder R, Studer UE (2003). Radical cystectomy for bladder cancer today—a homogeneous series without neoadjuvant therapy. J Clin Oncol.

[7] Bruins HM, Huang GJ, Cai J, Skinner DG, Stein JP, Penson DF (2009). Clinical outcomes and recurrence predictors of lymph node positive urothelial cancer after cystectomy. J Urol.

[8] Hautmann RE, de Petriconi RC, Volkmer BG (2010). Lessons learned from 1,000 neobladders: the 90-day complication rate. J Urol.

[9] Rodel C, Grabenbauer GG, Kuhn R, Papadopoulos T, Dunst J, Meyer M, Schrott KM, Sauer R (2002). Combined-modality treatment and selective organ preservation in invasive bladder cancer: long-term results. J Clin Oncol.

[10] Kaufman DS, Winter KA, Shipley WU, Heney NM, Chetner MP, Souhami L, Zlotecki RA, Sause WT, True LD (2000). The initial results in muscle-invading bladder cancer of RTOG 95-06: phase I/II trial of transurethral surgery plus radiation therapy with concurrent cisplatin and 5-fluorouracil followed by selective bladder preservation or cystectomy depending on the initial response. Oncologist.

[11] Shipley WU, Kaufman DS, Zehr E, Heney NM, Lane SC, Thakral HK, Althausen AF, Zietman AL (2002). Selective bladder preservation by combined modality protocol treatment: long-term outcomes of 190 patients with invasive bladder cancer. Urology.

[12] Kaufman DS, Winter KA, Shipley WU, Heney NM, Wallace HJ, Toonkel LM, Zietman AL, Tanguay S, Sandler HM (2009). Phase I-II RTOG study (99-06) of patients with muscle-invasive bladder cancer undergoing transurethral surgery, paclitaxel, cisplatin, and twice-daily radiotherapy followed by selective bladder preservation or radical cystectomy and adjuvant chemotherapy. Urology.

[13] Sternberg CN, Bellmunt J, Sonpavde G, Siefker-Radtke AO, Stadler WM, Bajorin DF, Dreicer R, George DJ, Milowsky MI, Theodorescu D, Vaughn DJ, Galsky MD, Soloway MS, Quinn DI (2013). ICUD-EAU International Consultation on Bladder Cancer 2012: Chemotherapy for Urothelial Carcinoma—Neoadjuvant and Adjuvant Settings. European urology.

[14] Babjuk M, Oosterlinck W, Sylvester R, Kaasinen E, Bohle A, Palou-Redorta J, Roupret M (2012). EAU guidelines on non-muscle-invasive urothelial carcinoma of the bladder, the 2011 update [Article in Spanish]. Actas Urol Esp.

[15] He XD, Wang ZP, Wei HY, Zhou Q, Wang DG, Tian JQ, Fu SJ, Rodriguez R (2009). Construction of urothelium-specific recombinant adenovirus and its inhibition in bladder cancer cell. Urol Int.

[16] Yang Y, Xu H, Shen J, Wu S, Xiao J, Xu Y, Liu XY, Chu L (2015). RGD-modifided oncolytic adenovirus exhibited potent cytotoxic effect on CAR-negative bladder cancer-initiating cells. Cell Death Dis.

[17] Melquist JJ, Kacka M, Li Y, Malaeb BS, Elmore J, Baseman AG, Hsieh JT, Koeneman KS (2006). Conditionally replicating adenovirus-mediated gene therapy in bladder cancer: an orthotopic *in vivo* model. Urol Oncol.

[18] Ramesh N, Ge Y, Ennist DL, Zhu M, Mina M, Ganesh S, Reddy PS, Yu DC (2006). CG0070, a conditionally replicating granulocyte macrophage colony-stimulating factor—armed oncolytic adenovirus for the treatment of bladder cancer. Clin Cancer Res.

[19] Wang H, Satoh M, Abe H, Sunamura M, Moriya T, Ishidoya S, Saito S, Hamada H, Arai Y (2006). Oncolytic viral therapy by bladder instillation using an E1A, E1B double-restricted adenovirus in an orthotopic bladder cancer model. Urology.

[20] Cook JL, Lewis AM (1984). Differential NK cell and macrophage killing of hamster cells infected with nononcogenic or oncogenic adenovirus. Science.

[21] Larson C, Oronsky B, Scicinski J, Fanger GR, Stirn M, Oronsky A, Reid TR (2015). Going viral: a review of replication-selective oncolytic adenoviruses. Oncotarget.

[22] Li L, Halaby MJ, Hakem A, Cardoso R, El Ghamrasni S, Harding S, Chan N, Bristow R, Sanchez O, Durocher D, Hakem R (2010). Rnf8 deficiency impairs class switch recombination, spermatogenesis, and genomic integrity and predisposes for cancer. J Exp Med.

[23] Yan J, Jetten AM (2008). RAP80 and RNF8, key players in the recruitment of repair proteins to DNA damage sites. Cancer Lett.

[24] Kolas NK, Chapman JR, Nakada S, Ylanko J, Chahwan R, Sweeney FD, Panier S, Mendez M, Wildenhain J, Thomson TM, Pelletier L, Jackson SP, Durocher D (2007). Orchestration of the DNA-damage response by the RNF8 ubiquitin ligase. Science.

[25] Mailand N, Bekker-Jensen S, Faustrup H, Melander F, Bartek J, Lukas C, Lukas J (2007). RNF8 ubiquitylates histones at DNA double-strand breaks and promotes assembly of repair proteins. Cell.

[26] Mallette FA, Mattiroli F, Cui G, Young LC, Hendzel MJ, Mer G, Sixma TK, Richard S (2012). RNF8- and RNF168-dependent degradation of KDM4A/JMJD2A triggers 53BP1 recruitment to DNA damage sites. Embo J.

[27] Mallette FA, Richard S (2012). K48-linked ubiquitination and protein degradation regulate 53BP1 recruitment at DNA damage sites. Cell Res.

[28] Wang B, Elledge SJ (2007). Ubc13/Rnf8 ubiquitin ligases control foci formation of the Rap80/Abraxas/Brca1/Brcc36 complex in response to DNA damage. Proc Natl Acad Sci U S A.

[29] Feng L, Chen J (2012). The E3 ligase RNF8 regulates KU80 removal and NHEJ repair. Nat Struct Mol Biol.

[30] Huen MS, Grant R, Manke I, Minn K, Yu X, Yaffe MB, Chen J (2007). RNF8 transduces the DNA-damage signal via histone ubiquitylation and checkpoint protein assembly. Cell.

[31] Vignard J, Mirey G, Salles B (2013). Ionizing-radiation induced DNA double-strand breaks: a direct and indirect lighting up. Radiother Oncol.

[32] Jackson SP, Bartek J (2009). The DNA-damage response in human biology and disease. Nature.

[33] Dietlein F, Reinhardt HC (2014). Molecular pathways: exploiting tumor-specific molecular defects in DNA repair pathways for precision cancer therapy. Clin Cancer Res.

[34] Ciccia A, Elledge SJ (2010). The DNA damage response: making it safe to play with knives. Mol Cell.

[35] Seo HK, Seo JB, Nam JK, Jeong KC, Shin SP, Kim IH, Lee SD, Lee SJ (2014). Development of replication-competent adenovirus for bladder cancer by controlling adenovirus E1a and E4 gene expression with the survivin promoter. Oncotarget.

[36] Nielsen ME, Smith AB, Meyer AM, Kuo TM, Tyree S, Kim WY, Milowsky MI, Pruthi RS, Millikan RC (2014). Trends in stage-specific incidence rates for urothelial carcinoma of the bladder in the United States: 1988 to 2006. Cancer.

[37] Oliver TG, Mercer KL, Sayles LC, Burke JR, Mendus D, Lovejoy KS, Cheng MH, Subramanian A, Mu D, Powers S, Crowley D, Bronson RT, Whittaker CA, Bhutkar A, Lippard SJ, Golub T (2010). Chronic cisplatin treatment promotes enhanced damage repair and tumor progression in a mouse model of lung cancer. Genes Dev.

[38] Bao S, Wu Q, McLendon RE, Hao Y, Shi Q, Hjelmeland AB, Dewhirst MW, Bigner DD, Rich JN (2006). Glioma stem cells promote radioresistance by preferential activation of the DNA damage response. Nature.

[39] Wang B, Matsuoka S, Ballif BA, Zhang D, Smogorzewska A, Gygi SP, Elledge SJ (2007). Abraxas and RAP80 Form a BRCA1 Protein Complex Required for the DNA Damage Response. Science.

[40] Takata M, Sasaki MS, Sonoda E, Morrison C, Hashimoto M, Utsumi H, Yamaguchi-Iwai Y, Shinohara A, Takeda S (1998). Homologous recombination and non-homologous end-joining pathways of DNA double-strand break repair have overlapping roles in the maintenance of chromosomal integrity in vertebrate cells. Embo J.

[41] Wu J, Huen MS, Lu LY, Ye L, Dou Y, Ljungman M, Chen J, Yu X (2009). Histone ubiquitination associates with BRCA1-dependent DNA damage response. Mol Cell Biol.

[42] Bonanno L, Costa C, Majem M, Sanchez JJ, Gimenez-Capitan A, Rodriguez I, Vergnenegre A, Massuti B, Favaretto A, Rugge M, Pallares C, Taron M, Rosell R (2013). The predictive value of 53BP1 and BRCA1 mRNA expression in advanced non-small-cell lung cancer patients treated with first-line platinum-based chemotherapy. Oncotarget.

[43] Rai R, Li JM, Zheng H, Lok GT, Deng Y, Huen MS, Chen J, Jin J, Chang S (2011). The E3 ubiquitin ligase Rnf8 stabilizes Tpp1 to promote telomere end protection. Nat Struct Mol Biol.

[44] Kim NW, Piatyszek MA, Prowse KR, Harley CB, West MD, Ho PL, Coviello GM, Wright WE, Weinrich SL, Shay JW (1994). Specific association of human telomerase activity with immortal cells and cancer. Science.

[45] Avilion AA, Piatyszek MA, Gupta J, Shay JW, Bacchetti S, Greider CW (1996). Human telomerase RNA and telomerase activity in immortal cell lines and tumor tissues. Cancer Res.

[46] Yan Z, Jiang J, Li F, Yang W, Xie G, Zhou C, Xia S, Cheng Y (2015). Adenovirus-mediated LRIG1 expression enhances the chemosensitivity of bladder cancer cells to cisplatin. Oncol Rep.

[47] Lu CS, Hsieh JL, Lin CY, Tsai HW, Su BH, Shieh GS, Su YC, Lee CH, Chang MY, Wu CL, Shiau AL (2015). Potent antitumor activity of Oct4 and hypoxia dual-regulated oncolytic adenovirus against bladder cancer. Gene Ther.

[48] Seo HK, Seo JB, Nam JK, Jeong KC, Shin SP, Kim IH, Lee SD, Lee SJ (2014). Development of replication-competent adenovirus for bladder cancer by controlling adenovirus E1a and E4 gene expression with the survivin promoter. Oncotarget.

[49] Babjuk M, Burger M, Zigeuner R, Shariat SF, van Rhijn BW, Comperat E, Sylvester RJ, Kaasinen E, Bohle A, Palou Redorta J, Roupret M (2013). EAU guidelines on non-muscle-invasive urothelial carcinoma of the bladder: update 2013. European urology.

[50] Burger M, Oosterlinck W, Konety B, Chang S, Gudjonsson S, Pruthi R, Soloway M, Solsona E, Sved P, Babjuk M, Brausi MA, Cheng C, Comperat E, Dinney C, Otto W, Shah J (2013). ICUD-EAU International Consultation on Bladder Cancer 2012: Non-Muscle-Invasive Urothelial Carcinoma of the Bladder. European urology.

[51] Mak RH, Hunt D, Shipley WU, Efstathiou JA, Tester WJ, Hagan MP, Kaufman DS, Heney NM, Zietman AL (2014). Long-term outcomes in patients with muscle-invasive bladder cancer after selective bladder-preserving combined-modality therapy: a pooled analysis of Radiation Therapy Oncology Group protocols 8802, 8903, 9506, 9706, 9906, and 0233. J Clin Oncol.

[52] Kim JH, Alfieri AA, Kim SH, Young CW (1986). Potentiation of radiation effects on two murine tumors by lonidamine. Cancer Res.

